# Supplementary material for: Associative properties of structural plasticity based on firing rate homeostasis in recurrent neuronal networks
Source: Sci Rep. 2018 Feb 28;8:3754. doi: 10.1038/s41598-018-22077-3 (PMC5830542; doi:10.1038/s41598-018-22077-3)
Supplement: Supplementary file 1 — Supplementary material [file 41598_2018_22077_MOESM1_ESM.pdf]

Associative properties of structural plasticity based on firing rate homeostasis in recurrent neuronal networks

Júlia V Gallinaro[1*], **Stefan Rotter**[1]

[1] Bernstein Center Freiburg & Faculty of Biology, University of Freiburg, Freiburg im Breisgau, Germany
[*] julia.gallinaro@bcf.uni-freiburg.de

Supplementary material

Change in average firing rate due to modulated external stimulation

We simulated a random recurrent network with static connections to estimate the expected change in average firing rate across the excitatory population due to modulated external stimulation, as described in the main text for the simulations of a generic visual cortex model. A network of N_E excitatory and N_I inhibitory LIF neurons was simulated. All connections were randomly chosen and static such that all neurons received C_E inputs from the excitatory population and C_I inputs from the inhibitory population. All neurons received Poissonian external input with rate ν_{ext} for the first 20 s of simulation, and external input with a rate modulated according to the input preferred orientation (PO) and the stimulus orientation (SO) for the last 20 s of the simulation. The input PO of all neurons was randomly chosen at the beginning of the simulation. A new SO was randomly chosen every 1 s (see visual stimulation protocol in the main text for details).

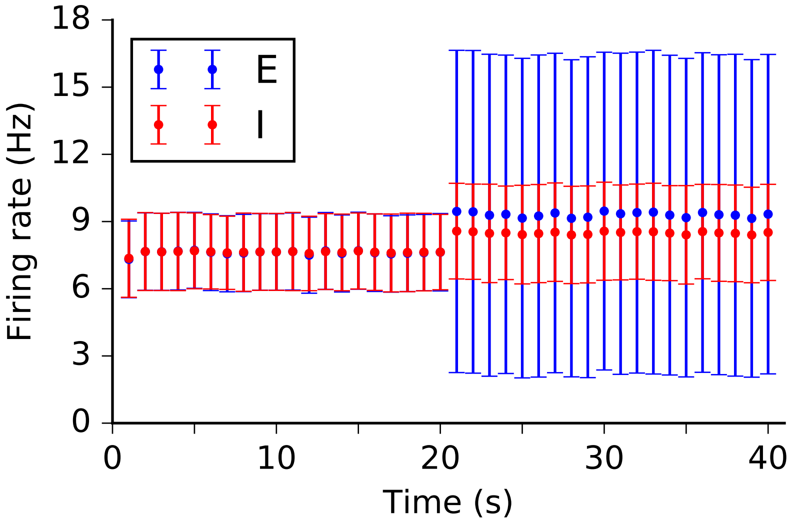

Figure 1. Change in average firing rate due to modulated external stimulation. Shown are mean and standard deviation of the firing rates (estimated from spike counts) of excitatory and inhibitory neurons for blocks of 1 s. The increase in standard deviation during stimulation is expected, since during stimulation neurons receive external input which is modulated according to the difference between their input PO and the SO.

Non-random features of networks grown with SP

Although we have been describing networks created with SP and unstructured external input as random, counting connectivity motifs between pairs of neurons shows a small bias towards bidirectional connections. Since multiple synapses between the same pair of neurons are allowed in the simulation, we consider a pair to be connected if there is at least one synapse between them. In a network where each individual contact between two neurons is a random variable from a Poisson distribution with parameter μ_c, the probability of a pair of neurons to have at least one synapse is $p_c = 1 - e^{-\mu_c}$. From this, we calculate the expected frequencies of motifs for the random network to be proportional to $(1 - p_c)^2$ for unconnected pairs, $2p_c(1 - p_c)$ for uni-directionally connected pairs, and p_c^2 for bidirectionally connected pairs.

The networks formed by SP without structured stimulation have slightly more bidirectional connections and fewer uni-directional connections than expected in a random network (Fig. 2A). These discrepancies are enhanced after feature-specific stimulation. Although these effects are rather weak, they go into the same direction as what has been observed in cortical networks[1,2]. Network models with spike-timing dependent plasticity (STDP), in contrast, have the opposite tendency. When bidirectional connections exist in a network, any asymmetric STDP rule will tend to strengthen the connection in one direction, while weakening the opposing one. Other plasticity rules based on STDP, like the triplet rule[3] and voltage-based STDP[4], however, have been shown to strengthen the connections in both directions of a bidirectional connection between neurons firing at high rates. An over-representation of bidirectional connections has also been shown by another computational model of multiple plasticity mechanisms, including a distance dependent kernel for the formation of new synapses[5]. Finally, Hoffmann & Triesch[6] recently showed that such an over-representation of bidirectional connections will necessarily emerge in networks in which connection probabilities are symmetric (as it is the case for an Erdős-Rényi random graph and related models) but non-homogeneous such that some pairs of neurons are more likely to be connected than others.

In our case, symmetric connection probabilities are clear from Fig. 5B in the main text and the non-uniformity follows from Fig. 2B and C. There is a strong positive correlation between in- and outdegree (Fig. 2B) that increases after modulated stimulation (Fig. 2C). Having a high correlation between the in- and outdegree of individual neurons in a network allows neurons to be classified into neurons with high connectivity (high in- and outdegree) and neurons with low connectivity (low in- and outdegree). Non-identical growth rules for pre- and postsynaptic elements, however, might lead to a different outcome. More studies with the SP model need to be performed to establish whether or not it is possible to produce networks with realistic numbers of bidirectional connections. Such studies could include combinations with other types of synaptic plasticity, the use of different homeostatic controllers for the synaptic elements, and the use of different target rate distributions.

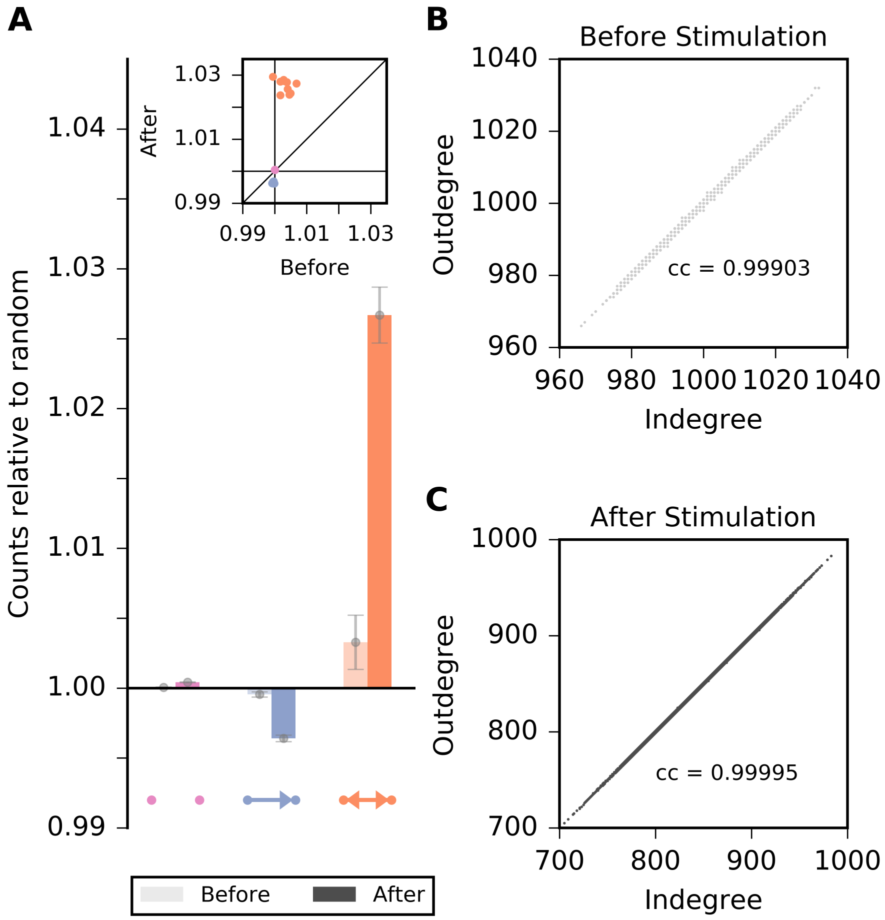

Figure 2. Non-random features of the networks grown with SP. (A) Relative frequency of pairwise connectivity motifs relative to what is expected for a random network with the same number of synapses. There is an over-representation of bidirectional connections and an under-representation of uni-directional connections that both become more prominent after oriented stimulation. Dots refer to the mean and bars to $\pm$ standard deviation across 10 independent simulation runs. *Inset:* Scatter plot of individual relative counts for the three different pairwise motifs before vs. after oriented stimulation, for 10 independent simulations. (B,C) Scatter plot of indegree and outdegree of individual excitatory neurons before and after stimulation. There is a correlation between indegree and outdegree that is also increased after stimulation.

Visual cortex simulation with target rates drawn from a distribution

We performed simulations in which target rates were drawn from a Gamma distribution with shape parameter $k = 64$ and scale parameter $\theta = 0.125$, yielding a distribution with mean 8 Hz and standard deviation 1 Hz, and found no significant difference in our main results. The equilibrium distribution of firing rates, and of the in- and outdegrees, had a larger variance when the target rates were drawn from a broader distribution. These results are in accordance with Pernice *et al.*[7], who demonstrated a positive correlation between the standard deviation of indegrees and the standard deviation of firing rates in simulated networks of LIF neurons of different topologies. A more detailed study using different target rate distributions for generating networks with the SP model would, therefore, lead to a better understanding of relationships between network structure and dynamics. A thorough study of the influence of the target rate distribution on the created networks were, however, beyond the scope of this paper.

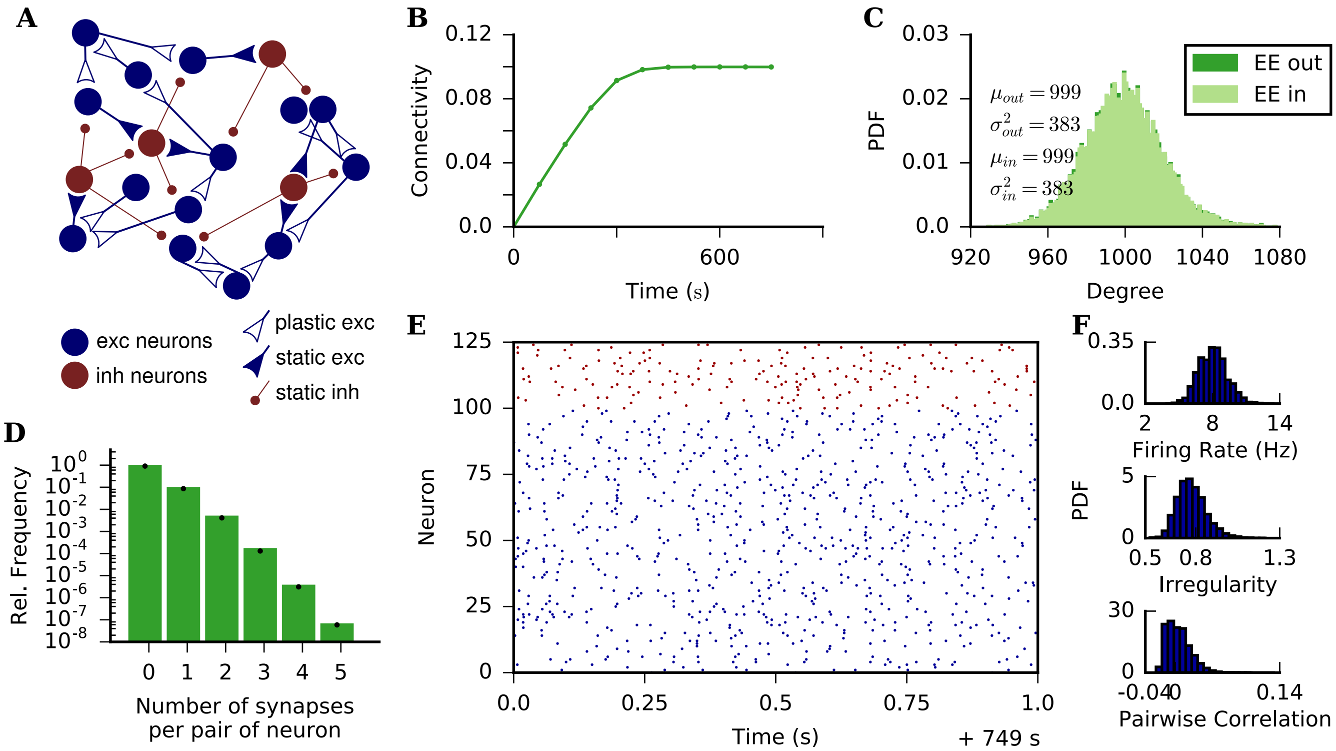

Figure 3. Same as Fig. 1 in the main text, but with target rates ρ drawn from a Gamma distribution.

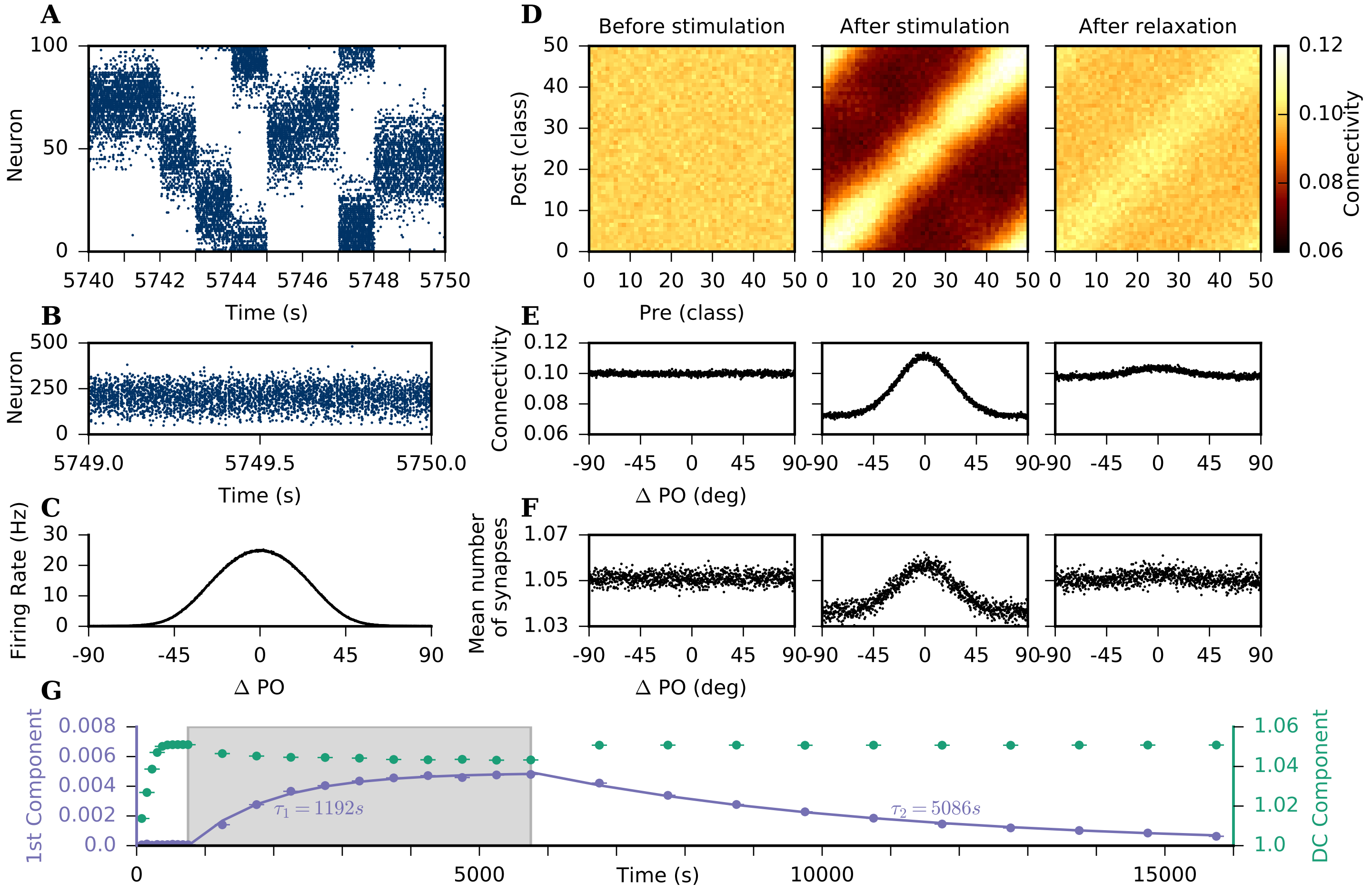

Figure 4. Same as Fig. 5 in the main text, but with target rates ρ drawn from a Gamma distribution.

Visual cortex simulation with decay of free elements

The SP model as it is implemented in NEST includes a decay of free synaptic elements. At each SP rewiring interval Δ_t, a percentage p of the synaptic elements which are not bound to a synapse are deleted. All the simulations in the main text do not consider a decay of free elements, and elements which are not bound to a synapse remain available as free elements to form new synapses. We also ran the same visual cortex simulation as described in the main text considering this decay of free elements with $p = 0.1$ and found no significant difference to our main results.

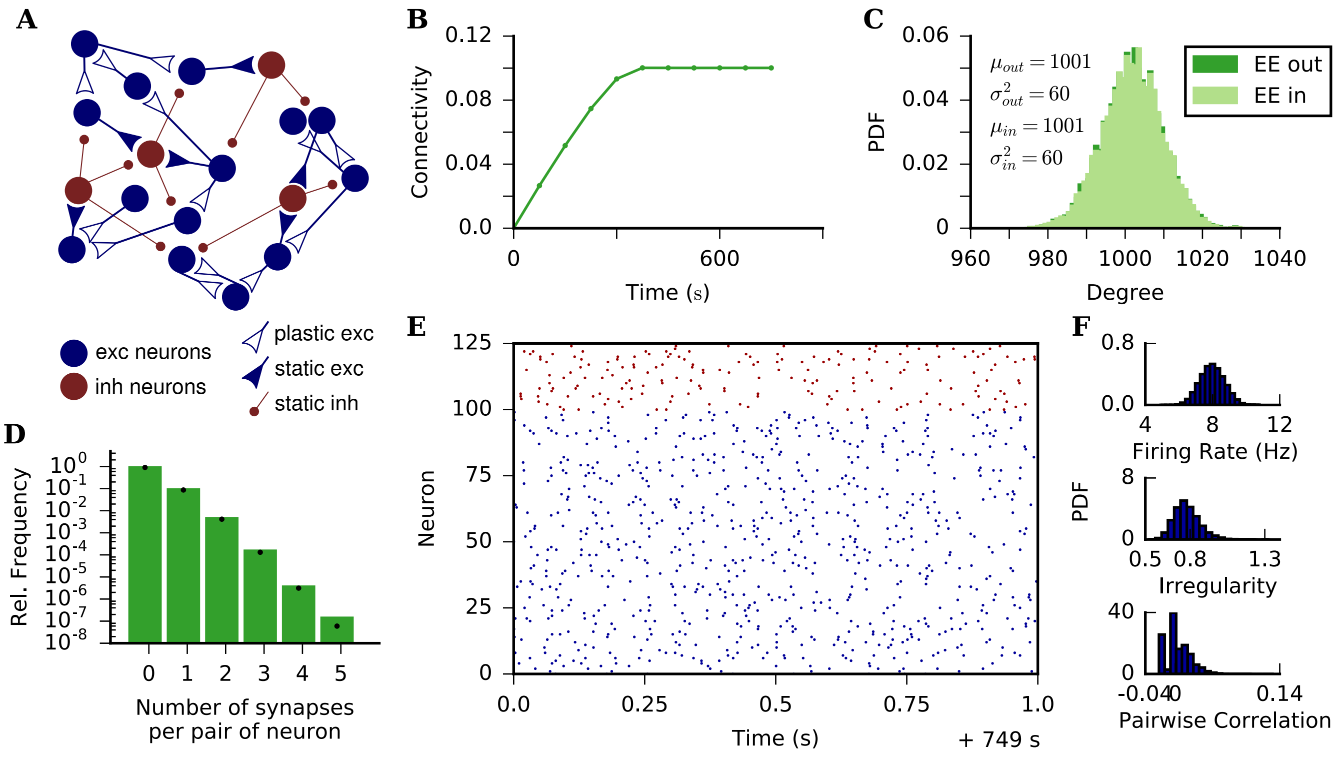

Figure 5. Same as Fig. 1 in the main text, but with decay of free elements. At each SP rewiring interval Δ_t, a percentage $p = 0.1$ of the synaptic elements not bound to a synapse are deleted.

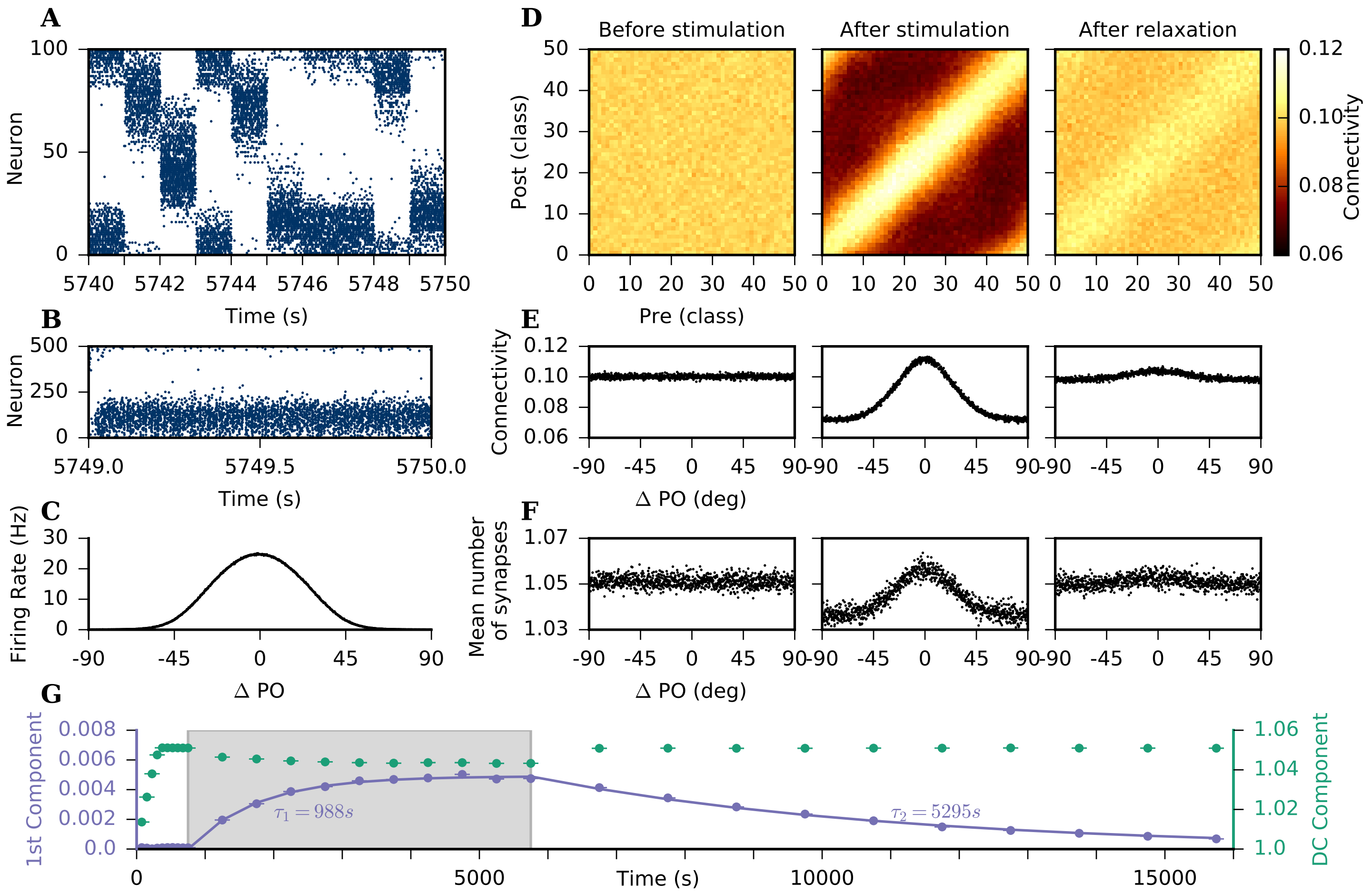

Figure 6. Same as Fig. 5 in the main text, but with decay of free elements. At each SP rewiring interval Δ_t, a percentage $p = 0.1$ of the synaptic elements not bound to a synapse are deleted.

Visual cortex simulation with target rates drawn from a distribution and decay of free elements

Finally, we ran simulations of visual cortex considering both the decay of free elements with a percentage $p = 0.1$ of free synaptic elements which are deleted after every SP rewiring interval Δ_t, and target rates ρ of excitatory neurons drawn from a Gamma distribution with shape parameter $k = 64$ and scale parameter $\theta = 0.125$, yielding a distribution with mean 8 Hz and standard deviation 1 Hz. We found no significant deviation to the main results described in the paper.

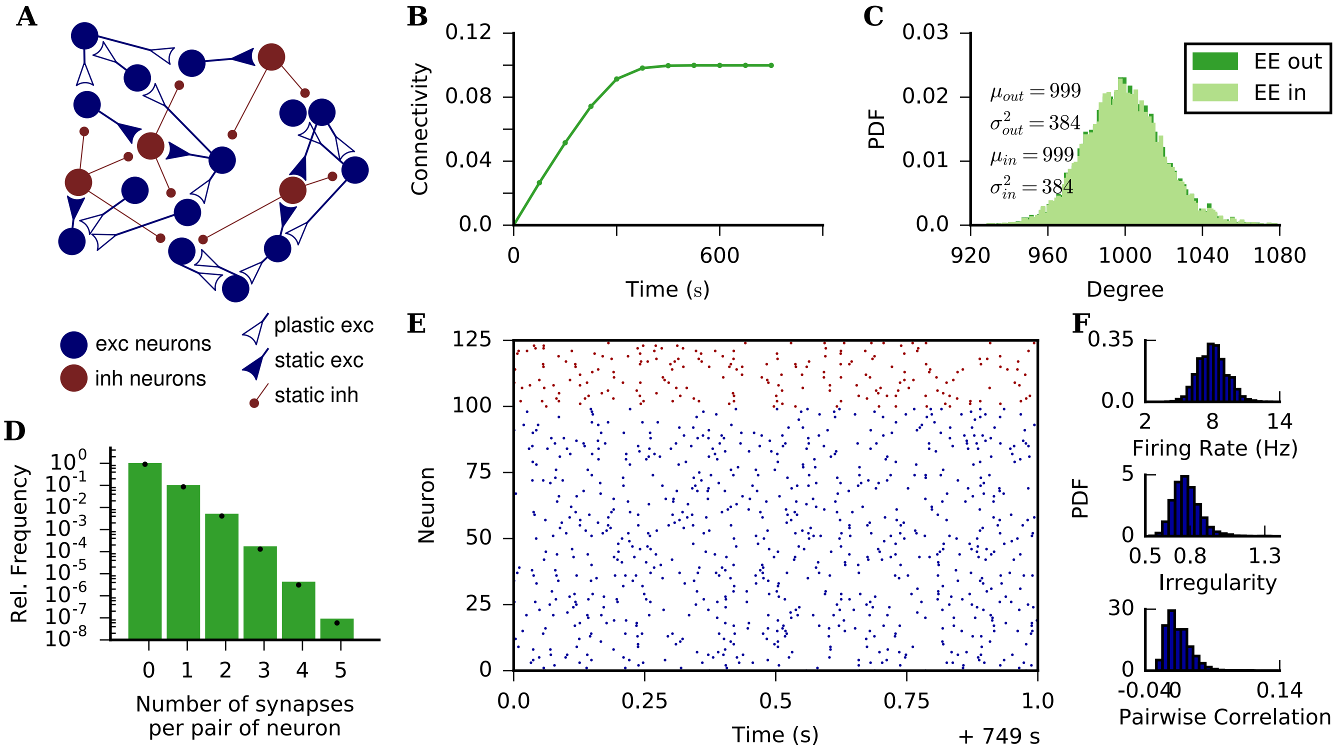

Figure 7. Same as Fig. 1 in the main text, but with decay of free elements and target rates ρ drawn from a Gamma distribution.

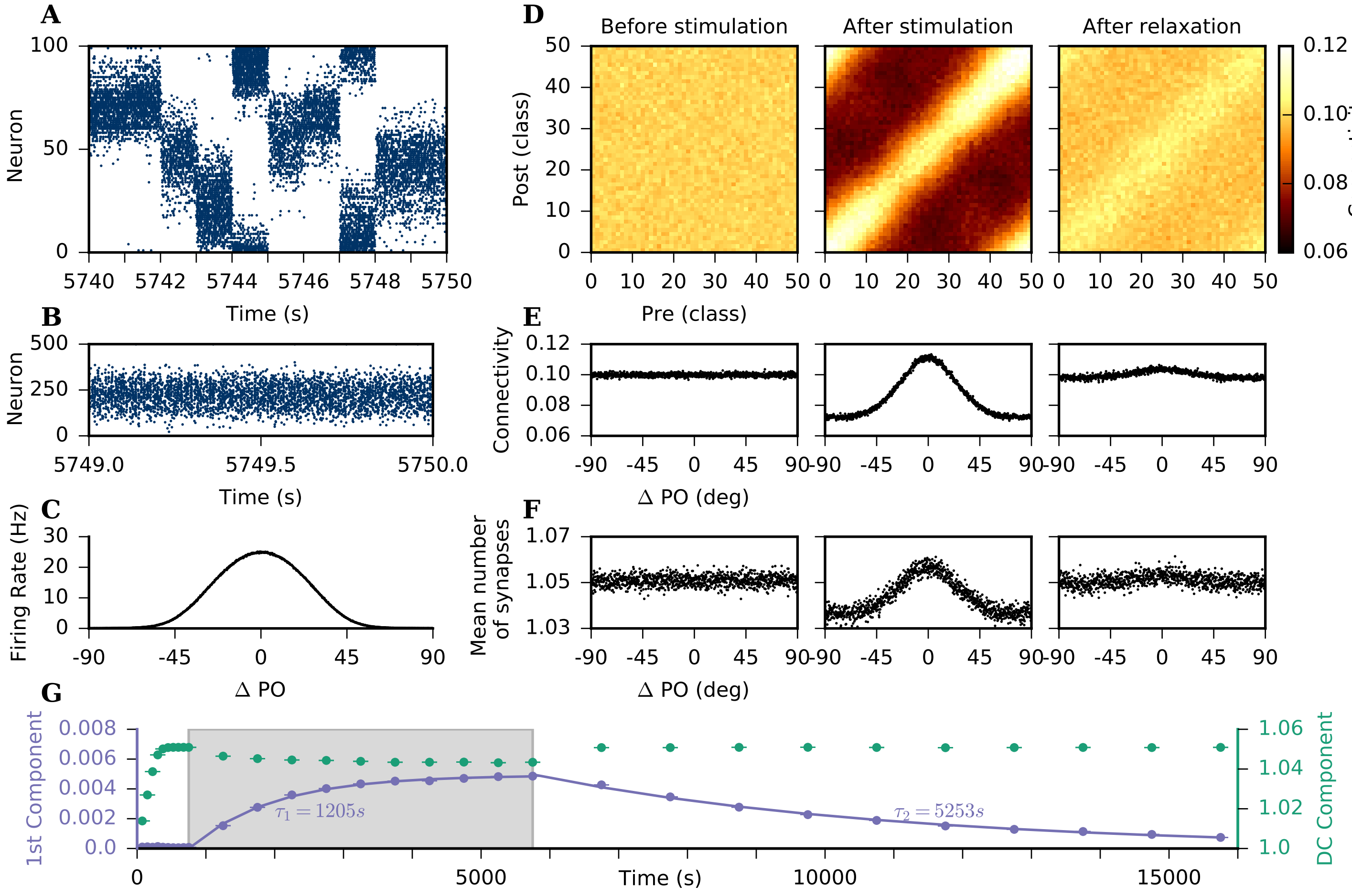

Figure 8. Same as Fig. 5 in the main text, but with decay of free elements and target rates ρ drawn from a Gamma distribution.

Modulation of mean number of synapses

Fig. 5G in the main text shows the time series of the connectivity modulation shown in Fig. 5E before, during and after modulated external stimulation. Based on the same data, we show here the modulation of the mean number of synapses (Fig. 5F).

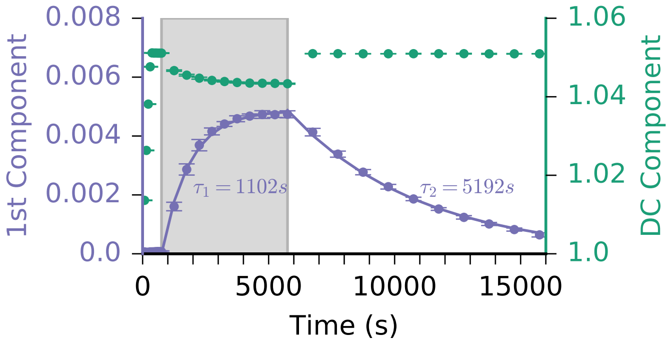

Figure 9. Same as Fig. 5G, but for the modulation of the mean number of synapses.

References

1. Song, S., Sjöström, P. J., Reigl, M., Nelson, S. & Chklovskii, D. B. Highly nonrandom features of synaptic connectivity in local cortical circuits. *PLOS Biol.* **3**, e68 (2005). URL https://www.ncbi.nlm.nih.gov/pubmed/15737062. DOI 10.1371/journal.pbio.0030068.

2. Perin, R., Berger, T. K. & Markram, H. A synaptic organizing principle for cortical neuronal groups. *Proc. Natl. Acad. Sci.* **108**, 5419–24 (2011). URL https://www.ncbi.nlm.nih.gov/pubmed/21383177. DOI 10.1073/pnas.1016051108.

3. Pfister, J.-P. & Gerstner, W. Triplets of spikes in a model of spike timing-dependent plasticity. *The J. Neurosci.* **26**, 9673–82 (2006). URL http://www.ncbi.nlm.nih.gov/pubmed/16988038. DOI 10.1523/JNEUROSCI.1425-06.2006.

4. Clopath, C., Büsing, L., Vasilaki, E. & Gerstner, W. Connectivity reflects coding: a model of voltage-based STDP with homeostasis. *Nat. Neurosci.* **13**, 344–352 (2010). URL https://www.ncbi.nlm.nih.gov/pubmed/20098420. DOI 10.1038/nn.2479.

5. Miner, D. & Triesch, J. Plasticity-Driven Self-Organization under Topological Constraints Accounts for Non-random Features of Cortical Synaptic Wiring. *PLOS Comput. Biol.* **12**, e1004759 (2016). URL www.ncbi.nlm.nih.gov/pubmed/26866369. DOI 10.1371/journal.pcbi.1004759.

6. Hoffmann, F. Z. & Triesch, J. Nonrandom network connectivity comes in pairs. *Netw. Neurosci.* **1**, 31–41 (2017). URL http://www.mitpressjournals.org/doi/10.1162/NETN{_}a{_}00004. DOI 10.1162/NETN_a_00004.

7. Pernice, V., Deger, M., Cardanobile, S. & Rotter, S. The relevance of network micro-structure for neural dynamics. *Front. Comput. Neurosci.* **7**, 72 (2013). URL https://www.ncbi.nlm.nih.gov/pubmed/23761758. DOI 10.3389/fncom.2013.00072.
